# The Diseases of Childhood (Medical)

**Published:** 1894-06

**Authors:** 


*The Diseases of Childhood (Medical).* By H. BRYAN DONKIN, M.D. Pp. xiv., 433. London: CHARLES GRIFFIN AND COMPANY, LIMITED. 1893.

If a guarantee for the vitality of a subject is to be found in the quantity of its literature, then the study of disease in children, or, as it is fashionable to call it, the science of pediatrics, is very full of life indeed.

At short intervals (we had almost written every month or so) a work is presented to the medical profession purporting to be written by some authority upon the subject, and treating of those diseases generally considered to be peculiar to children. These productions vary in size and contents: in size from the small hand-book or student's guide to the overwhelming encyclopædia in many volumes; and regarding their contents may be roughly divided into two classes,—first and most numerous, those belonging to the "paste-and-scissors" division, mere repetitions of accepted theory and practice, serving no purpose except to multiply needlessly medical books and advertise the "authors;" and second, those works which are useful guides indeed, books giving the results of years of hard clinical study, *original* ideas and research, and are valuable and welcome additions to our knowledge of disease in children. To this latter class the book before us undoubtedly belongs. The author, who has the advantage which the double position of physician to a general *and* a children's hospital gives him, whilst treating his subject with that fulness of detail and accuracy of judgment which his position as a specialist places at his command, adds to these qualities a breadth of view and a general grasp of the subject in relation to the science of medicine as a whole which are often wanting in works written on special branches of our art. For the masterly manner in which they are handled, we would particularly commend attention to the following chapters: "Infantile Wasting," "Affections of the Fauces," "Intestinal Obstruction," and "Spasmodic Disorders."

With due regard to Dr. Donkin's preface, we do not think that any hand-book on disease in children intended for students can be considered complete without a chapter on Smallpox, a subject which in these days of advancing "public health" is of increasing interest and importance: and we were hardly prepared to find in the treatment of constipation of young children, injections of glycerine entirely ignored; or the management of acute tonsillitis summed up without any mention being made of small doses of tincture of aconite. The book however, taken as a whole, is one we can heartily recommend. It is well printed; a good index and an appendix on the "Periods of Incubation and Contagiousness of certain Infectious Diseases" add to its value.

